# Retrospective Evaluation of Outcome in Dogs With Appendicular Osteosarcoma Following Hypofractionated Palliative Radiation Therapy With or Without Bisphosphonates: 165 Cases (2010–2019)

**DOI:** 10.3389/fvets.2022.892297

**Published:** 2022-05-10

**Authors:** Beck Ringdahl-Mayland, Douglas H. Thamm, Tiffany W. Martin

**Affiliations:** ^1^Department of Clinical Sciences, College of Veterinary Medicine and Biomedical Sciences, Colorado State University, Fort Collins, CO, United States; ^2^Flint Animal Cancer Center, Colorado State University, Fort Collins, CO, United States; ^3^Department of Environmental and Radiological Health Sciences, College of Veterinary Medicine and Biomedical Sciences, Colorado State University, Fort Collins, CO, United States

**Keywords:** survival, bone, cancer, radiotherapy, dog

## Abstract

**Objective:**

To report the survival times in dogs that received a standardized palliative-intent radiation therapy (RT) protocol for the treatment of canine appendicular osteosarcoma (OSA), alone or in combination with bisphosphonates (BPs), and to determine whether the addition of BPs affects survival. A secondary objective was to identify prognostic features that may influence outcome in dogs undergoing treatment.

**Design:**

Retrospective case series.

**Materials and Methods:**

Dogs with presumed or confirmed OSA of the appendicular limb treated with daily hypofractionated RT (8 Gy x 2) at the Flint Animal Cancer Center between 2010 and 2019 were evaluated retrospectively. Clinical data were abstracted from the medical records, and adjuvant therapies were noted. Outcome was assessed using medical records and electronic follow up.

**Results:**

One hundred and sixty-five dogs were included. Sixty-eight dogs received BPs as a part of their palliative-intent treatment. The median survival time from first RT treatment to death was not significantly different between groups (119 vs. 109 days for BP and non-BP groups, respectively, *p* = 0.758). Only age (>9 years) was found to be prognostic in this population (*p* = 0.031). Factors that were not found to be associated with survival time included BP drug type, timing of BP administration, tumor location, weight, breed, sex, time to treatment, concurrent administration of chemotherapy, and salvage amputation.

**Conclusions:**

This study suggests no difference in outcome for dogs treated with and without BPs in addition to hypofractionated RT. Prospective studies are needed to determine if the addition of BPs to hypofractionated RT leads to an improved quality of life in dogs undergoing palliative-intent treatment for OSA.

## Introduction

Osteosarcoma (OSA) is the most common primary bone tumor of dogs, and commonly occurs in the bones of the appendicular skeleton. This tumor is reported to account for up to 85% of tumors of the skeleton, and up to 98% of primary bone tumors of the appendicular skeleton (1, 2). Affected dogs are most commonly middle-aged to older, with most dogs diagnosed between 7 and 9 years of age (1, 2). Large to giant breeds are at an increased risk of developing appendicular OSA, with Greyhounds, Rottweilers, and Great Danes among the breeds that are overrepresented (1, 3).

Definitive-intent treatment of dogs with appendicular OSA currently consists of limb amputation or limb-sparing procedures (limb sparing surgery or stereotactic body radiation therapy) for local tumor control, followed by adjuvant chemotherapy to delay metastasis (1, 4). Dogs may not be considered candidates for definitive-intent treatment for many reasons, including treatment-dependent risk, owner-related reasons, or presence of metastatic disease. For these dogs, palliative-intent treatment is commonly pursued to alleviate tumor-related clinical signs and improve quality of life. This may be accomplished with a combination of radiation therapy (RT), oral analgesics, and bisphosphonate (BP) drugs.

Several protocols exist for palliative-intent RT, with fractions of 6 to 10 Gy commonly administered on a schedule varying from weekly to consecutively within a 24 h period (5–10). These protocols have been associated with significant pain relief and/or improved quality of life in ~75–90% of dogs (2, 5–9). Several studies have also evaluated the concurrent administration of chemotherapy with palliative-intent RT. While some studies have suggested that the addition of chemotherapy may improve survival times, the majority have identified no significant additional benefit (7, 8, 11).

Bisphosphonates are synthetic analogs of pyrophosphate with a high affinity for bone mineral (12). This class of drugs preferentially bind to hydroxyapatite crystals within bone and have been found to inhibit osteoclast activity (12). Newer aminobisphosphonates, such as zoledronate (ZOL) and pamidronate (PAM), may also induce apoptosis of osteoclasts, but this is not necessary for their antiresorptive properties (12, 13). This class of drugs has become widely used in human and veterinary medicine for the treatment of osteoclast-mediated disease, hypercalcemia, and osteolysis secondary to malignant disease, including myeloma-related disease, bone metastases, and primary bone lesions (1, 12, 13). Side effects of this class of drugs are generally considered rare. The most clinically important adverse effect of BP administration in dogs is acute renal insufficiency. A recent retrospective evaluation identified azotemia in ~8% of dogs following administration of ZOL, but a causal relationship could not be identified (14). Other reported abnormalities include electrolyte disturbances and osteonecrosis, the latter of which is clinically important in human medicine, but has thus far only been reported in one dog (12–15).

Bisphosphonates and palliative-intent RT are commonly administered to dogs for the purpose of relieving pain associated with presumed or confirmed OSA of the appendicular skeleton. Several preclinical studies have suggested a possible benefit from the coadministration of these treatments, such as improved bone strength and radiosensitization in mouse models of tumor-induced osteolysis (16, 17). More recently, *in vitro* work with canine OSA cells has identified a possible antagonistic interaction when radiation and BPs are administered concurrently, and a possible benefit to delayed administration of BPs following radiation (18, 19). Unfortunately, despite their common clinical use in the palliative-intent treatment of canine OSA, their use is infrequently reported in the literature. The combination of PAM with palliative-intent RT has been reported in two prior studies, with one suggesting that coadministration was safe but did not clearly result in improved pain control, and the other suggesting that inclusion of PAM led to a significant reduction in median survival time (MST) (10, 20). There are no studies reporting the combination of ZOL with palliative-intent RT in canine OSA.

The aim of this retrospective study was to report the survival times in dogs that received a standardized palliative-intent RT protocol for the treatment of canine appendicular OSA, alone or in combination with BPs, and to determine whether the addition of BPs (PAM or ZOL) affected survival. A secondary aim was to identify prognostic features that may influence outcome in dogs undergoing treatment.

## Materials and Methods

The radiation oncology treatment database at the Colorado State University Veterinary Teaching Hospital was queried for dogs undergoing a palliative-intent RT protocol for treatment of bone lesions with a standardized hypofractionated protocol (8 Gy x 2) administered as consecutive daily treatments between February 2010 and September 2019. For inclusion, dogs needed to have a solitary appendicular lesion that was confirmed as OSA on histopathology or cytology, or was presumed to represent OSA based on imaging characteristics as interpreted by an ACVR board-certified veterinary radiologist. Additionally, dogs needed to have completed at least one RT protocol. Dogs were excluded if they had a primary scapular lesion, or if they had previously undergone a definitive-intent local treatment. Dogs with presumptive or confirmed metastatic disease or pathologic fracture at the time of treatment were not excluded from this study.

Medical records were reviewed, and follow-up data was collected from referring veterinarians. The signalment, body weight, primary diagnosis, affected bone, concurrent treatments, date of first visit, dates of RT treatments, dates of BP treatments, and date of death were recorded for each case. A diagnosis of OSA was considered definitive if there was cytological or histological confirmation from a board-certified clinical or anatomic pathologist. BPs were considered to be administered prior to RT if administered on any date before the first RT fraction, concurrent if administered on the same date as any RT fraction, and after if administered on any date following the second RT fraction. As BPs are commonly administered multiple times, these categories were not treated as if they were discrete, such that a dog receiving BPs on the same date as RT fraction 1 and 4 weeks later would be categorized as having received BPs concurrent and after RT.

Categorical variables were counted, and proportions used to summarize the data. Continuous variables were reported as medians and ranges because they were not normally distributed. Signalment and tumor location were evaluated between groups. Age and weight were compared between cohorts using a two-tailed Mann-Whitney test. Sex, breed, and tumor location were compared between cohorts using a two-tailed chi-square or Fisher exact test. Survival time was calculated as the time between the first day of RT treatment and death. Dogs were censored at the date when they were lost to follow-up, and were censored at time of death only if a necropsy performed by a board-certified anatomical pathologist identified a cause of death that was unrelated to OSA or treatments administered. Kaplan-Meier survival curves were created and median survival times were estimated for the populations of interest. A Cox proportional hazards model and logrank analysis (Prism 9, Graph Pad Software, La Jolla, California) were used to fit the variables age, weight, breed, sex, tumor location, administration of BP, concurrent administration of chemotherapy, and amputation following treatment to survival models while accounting for censoring. Dogs within the group receiving BPs were also evaluated based on whether they received ZOL or PAM, and whether BPs were administered concurrently with RT or not. A *p*-value of <0.05 was considered statistically significant.

## Results

A database search revealed 211 patients that received the hypofractionated palliative RT protocol of interest (8 Gy x 2) between the dates of interest. Ten patients received this protocol for treatment of metastatic lesions following definitive-intent treatments, 10 patients had more than one lesion that was treated concurrently, 8 patients had treatment of axial skeleton lesions, 6 patients had primary scapular lesions, 6 patients did not complete the protocol, 5 patients were part of a clinical trial where it could not be determined if they did or did not receive a BP, and 1 patient had treatment of a metastatic lymph node. This resulted in the exclusion of 46 cases, for a total of 165 patients that were available for further evaluation. Palliative RT was selected for treatment due to owner preference, concurrent metastatic disease, orthopedic or neurologic disease, and other comorbidities, although the specific reason was not clear for each dog based on the available records. Palliative RT was delivered utilizing 3-dimensional conformal radiation therapy (3D-CRT) or manual set up with parallel opposed fields with 6 or 10 MV photons on a Varian Trilogy Linear Accelerator (Varian Medical Systems, Inc., Palo Alto, CA).

A summary of the presenting signalment and tumor location for all dogs can be found in Table 1. The median age at initial admission for this population was 9 years old (range: 3–14) and weight was 39.0 kg (range: 10–93.6 kg). The median age of dogs who received bisphosphonates (BP+) was 9 years old (range: 3–14) and the median age of dogs who did not receive bisphosphonates (BP–) age was 10 years old (range: 4–14). These groups were significantly different from each other (*p* = 0.03). The median BP+ weight was 43.8 kg (range: 10–93.6 kg) and the median BP– weight was 36.9 kg (range: 12.3–67 kg). These groups were significantly different from each other (*p* = 0.0005). Patients included 88 neutered males, 71 spayed females, 4 intact males, and 2 intact females. No significant differences were found between sex distribution in each group. Thirty six breeds were represented: Labrador Retriever (*n* = 24), Golden Retriever (18), Rottweiler (13), Greyhound (12), Saint Bernard (10), Great Dane (9), Mastiff (7), Great Pyrenees (5), Bernese Mountain Dog (4), Australian Shepherd (3), Doberman (3), Newfoundland (2), English Bulldog (2), Rhodesian Ridgeback (2), German Shepherd Dog (2), Anatolian Shepherd (2), one of each of the following German Shorthaired Pointer, German Wirehaired Pointer, Irish Wolfhound, Kerry Blue Terrier, Pointer, Malamute, Cocker Spaniel, Belgian Sheepdog, Corgi, Olde English Sheepdog, Bullmastiff, Poodle, Staffordshire Terrier, Dogue de Bordeaux, Bouvier des Flandres, Airedale, Weimaraner, Husky, Irish Setter, and 28 mixed breed dogs. Only a significant difference in the distribution of Saint Bernards was present between groups (*p* = 0.017).

**Table 1:** Signalment and tumor location information for all dogs, and by group.

**Characteristic**		**All (*n* = 165)**	**BP+ (*n* = 68)**	**BP– (*n* = 97)**	***p*-value**
Age (years)	Median (range)	9 (3–14)	9 (3–14)	10 (4–14)	0.03^*^
Weight (kg)	Median (range)	39 (10–93.6)	43.8 (10–93.6)	36.9 (12.3–66.5)	0.0005^*^
Sex	Male intact	4	3	1	0.31
	Female intact	2	0	2	0.51
	Male neutered	88	33	55	0.34
	Female spayed	71	32	39	0.43
Breed	Mixed breed	28	8	20	0.15
	Labrador retriever	24	7	17	0.26
	Golden retriever	18	8	10	0.8
	Rottweiler	13	6	7	0.77
	Greyhound	12	3	9	0.36
	Saint Bernard	10	8	2	0.017^*^
	Great Dane	9	6	3	0.16
	Mastiff	7	3	4	>0.99
	Great Pyrenees	5	3	2	0.4
	Bernese mountain dog	4	2	2	>0.99
	Australian shepherd	3	1	2	>0.99
	Doberman pinscher	3	1	2	>0.99
	Newfoundland	2	1	1	>0.99
	English bulldog	2	1	1	>0.99
	Rhodesian ridgeback	2	1	1	>0.99
	German shepherd	2	0	2	0.51
	Anatolian shepherd	2	1	1	>0.99
	Other (1 each)	19	8	11	>0.99
Location	Forelimb	127	55	72	0.35
	Hindlimb	38	13	25	0.35
	Radius	65	27	38	>0.99
	Humerus	59	28	31	0.25
	Tibia	20	7	13	0.63
	Femur	17	6	11	0.8
	Ulna	3	0	3	0.27
	Fibula	1	0	1	>0.99

Statistically significant differences between BP+ and BP– groups are denoted by an asterisk (^*^).

A majority of dogs had a tumor of the forelimb (*n* = 127; 77%), with a minority of dogs having a tumor affecting the hindlimb (*n* = 38; 23%). Sixty five patients had tumors of the radius (39%), 59 had tumors of the humerus (36%), 20 had tumors of the tibia (12%), 17 had tumors of femur (10%), 3 had tumors of the ulna (2%), and 1 had a tumor of the fibula (<1%). There was no significant difference in the distribution of tumor location between groups. Diagnosis of OSA was definitive in 55 dogs (33%) and presumptive in 110 dogs (67%). The MST for dogs with a definitive diagnosis of OSA was 119 days, and the MST for dogs with a presumptive diagnosis was 107 days. There was no significant different in MST between these groups (*p* = 0.73). A majority of dogs underwent only one RT course (*n* = 135; 82%), with the rest undergoing more than one RT course (*n* = 30; 18%). Of the patients undergoing multiple courses, 27 underwent two (90%) and 3 underwent three (10%). Seventeen of the BP+ dogs underwent multiple RT courses (25%), and 13 of the BP– dogs underwent multiple courses (13%). This difference was not significant (*p* = 0.057). Two of the dogs undergoing three courses were BP+, and one was BP–. The median time from the date of initial presentation to first RT treatment was 3 days (range: 0–147 days). For dogs undergoing more than one course of RT, the median time from completion of the first course to initiation of the second course was 84 days (range: 41–340 days). For the three dogs who underwent 3 courses of RT, the number of days between completion of the second course and initiation of the third course was 135 days (BP+), 140 days (BP+), and 252 days (BP–).

In addition to RT, 68 dogs received BP (BP+; 41%) while 97 dogs did not receive BP (BP–; 59%). Of the BP+ dogs, 37 were administered PAM (54%) and 31 were administered ZOL (46%). Fifty-two dogs received BPs concurrently with their hypofractionated RT treatment (76%), 12 received BPs prior to treatment (18%), and 22 received BPs following treatment (32%). The majority of dogs receiving BPs had a dose administered within 30 days of treatment (*n* = 63; 93%).

Thirty seven dogs (22%) received chemotherapy in addition to RT at some point during their care. Two dogs had chemotherapy initiated prior to RT (5%), 26 dogs received chemotherapy as concurrent treatment (70%), and 9 dogs received chemotherapy following completion of RT as a form of rescue therapy due to evidence of newly onset or progressive metastatic disease (25%). Chemotherapy protocols varied widely between patients, with 6 patients receiving more than one protocol over the course of their care. Protocols utilized included: single agent carboplatin (*n* = 22), metronomic cyclophosphamide (7), toceranib phosphate (5), toceranib phosphate with high dose losartan (5), toceranib phosphate with metronomic cyclophosphamide (3), carboplatin with high dose losartan (2), and single agent doxorubicin (1).

Nineteen dogs (11%) had documented evidence of metastatic disease prior to initiating RT. This included 10 BP+ dogs (15%), and 9 BP– dogs (9%). All dogs with evidence of metastatic disease in the BP+ group had pulmonary involvement. The BP+ group included 2 dogs who were censored due to being lost to follow up. The median survival within the BP+ group with censoring was 83.5 days (range: 35–203 days). Five dogs within the BP+ group received chemotherapy. Three dogs received toceranib phosphate with high dose losartan, and 2 dogs received single agent carboplatin. The dogs with evidence of metastatic disease in the BP– group included one dog with lymph node metastasis, while all other dogs had pulmonary involvement. The median survival within the BP– group was 57 days (range: 16–497 days). Three dogs within the BP– group received chemotherapy. One dog received toceranib phosphate, 1 dog received toceranib phosphate with high dose losartan, and 1 dog received metronomic cyclophosphamide. The median survival for all non-censored dogs with evidence of metastatic disease (BP+ and BP–) receiving chemotherapy was 118 days (range = 16–497 days).

Twelve dogs (7%) were reported to have an amputation performed at some point following RT. Ten of the dogs reported to have an amputation were BP– and 2 were BP+ (*p* = 0.073). Complete records related to this procedure were not available for all dogs. The date of amputation was available for 8 dogs, and the median time from completion of the first course of RT to amputation was 56 days (range: 14–296 days). Of the records that were available for review, 6 identified a confirmed pathologic fracture as the reason for amputation (50%). Of the dogs with a confirmed pathologic fracture, all were BP–. Five of 6 dogs with a confirmed pathologic fracture had a known date of amputation; the median time from completion of the first course of RT to amputation for this subset was 48 days (range: 14–296 days).

**Figure 1:**
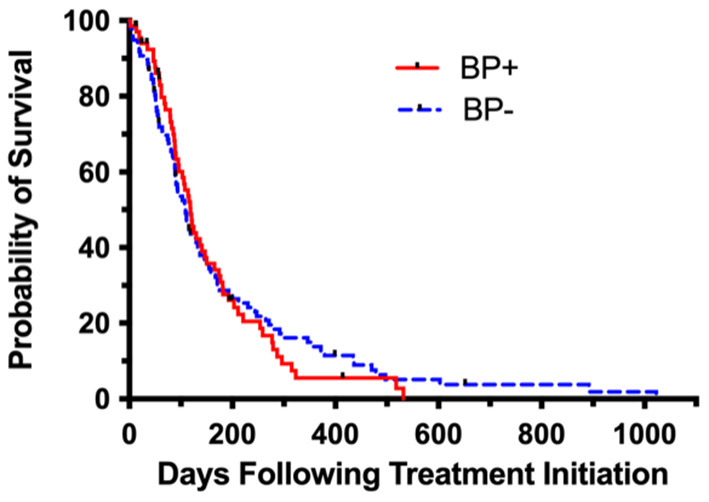
Kaplan-Meier survival curve analysis of BP+ (bisphosphonate) vs. BP– (no bisphosphonate) dogs. The median survival time of BP+ dogs was not significantly different than the median survival time for BP– dogs (119 vs. 109 days and *p* = 0.758).

Regardless of other therapies, dogs that received BPs in their treatment had a MST of 119 days compared to 109 days for dogs that did not (Figure 1). Survival time was not affected by the addition of BPs [*p* = 0.758, HR (95% CI) = 1.05 (0.76–1.47)] (Table 2). When considering dogs that received BPs as a part of their treatment, dogs that received PAM had a MST of 119 days compared to 121 days for dogs that received ZOL. Survival was not affected by the BP drug that was administered [*p* = 0.742, HR (95% CI) = 1.09 (0.65–1.83)]. Dogs that received BPs concurrent with RT had a MST of 114 days compared to 147 days for those who received BPs prior to and/or following RT. Survival was not affected by the concurrent administration of BPs with treatment [*p* = 0.231, HR (95% CI) = 0.72 (0.41–1.23)].

**Table 2:** Median survival time for evaluated variables.

	**Factor**	**MST**	***p*-**
		**(days)**	**value**
All – bisphosphonates administered	Yes (BP+)	119	0.758
	No (BP–)	109	
BP – drug administered	Pamidronate	119	0.742
	Zoledronate	121	
BP – timing of administration	Concurrent with RT	114	0.231
	Not concurrent with RT	147	
All – tumor location	Forelimb	118	0.239
	Hindlimb	85	
All – weight (kg)	<39	103	0.433
	≥39	119	
All – age (years)	≥9	125	0.031^*^
	>9	96	
All – breed	Giant breeds	119	0.233
	Non-giant breeds	109	
All – sex	Male	116	0.741
	Female	114	
All – time to treatment (days)	≤ 7	116	0.929
	>7	111	
All – concurrent chemotherapy	Yes	116	0.605
	No	111	
All – amputation performed after RT	Yes	203	0.108
	No	111	

*Variables with the prefix “All” denote categories in which the factor was evaluated for the entire population (n = 165). Variables with the prefix “BP” denote categories in which the factor was only evaluated for BP+ dogs (n = 68)*.

Statistically significant differences between groups are denoted by an asterisk (^*^).

Dogs that were ≤9 years of age at the time of first presentation had a MST of 125 days compared to 96 days for dogs that were >9 years of age. Survival was negatively affected by increased age [*p* = 0.031, HR (95% CI) = 0.71 (0.51–0.98)]. Other signalment and clinical features were not found to affect survival, including: weight < 39 kg vs. ≥39 kg [*p* = 0.433, HR (95% CI) = 1.14 (0.82–1.57)], non-giant breeds vs. giant breeds [*p* = 0.233, HR (95% CI) = 0.8 (0.53–1.20)], female dogs vs. male dogs [*p* = 0.741, HR (95% CI) = 0.95 (0.68–1.31)], and forelimb vs. hindlimb tumor location [*p* = 0.239, HR (95% CI) = 0.8 (0.53–1.20)].

No treatment-related considerations were identified as significantly influencing survival. Dogs that initiated RT within 7 days of their initial presentation had a MST of 116 days compared to 111 days for dogs that started treatment more than 7 days after their initial presentation [*p* = 0.929, HR (95% CI) = 0.98 (0.68–1.42)]. Dogs that did not receive chemotherapy concurrently with RT had a MST of 111 days compared to 116 days for dogs that did receive concurrent chemotherapy [*p* = 0.605, HR (95% CI) = 1.13 (0.72–1.78)]. Dogs that did not have an amputation performed following RT had a MST of 111 days, while dogs that did have an amputation had a MST of 203 days [*p* = 0.108, HR (95% CI) = 1.64 (0.99–2.70)].

## Discussion

Despite hypofractionated RT and BPs being common interventions in the palliative-intent treatment of canine OSA, there is a dearth of information available about the outcome following these interventions. In this retrospective case series, the MST of dogs that received BPs in addition to hypofractionated RT was not significantly different from dogs that did not receive BPs, with these groups having survival times of ~4 months from the initiation of RT. This finding is in contrast to a prior study which suggested a significantly worse outcome with the inclusion of BPs (8). In this prior study, which was previously the only report of outcome in dogs receiving BPs with palliative-intent RT, the inclusion of PAM led to a statistically significant reduction in MST from 247 to 124 days (8). A similar reduction in survival time was not observed in the current study, but the MST was similar to that reported for all dogs receiving PAM. This discrepancy could be related to several differences in this contemporary cohort. The previous study included dogs that received multiple (at least 4) different hypofractionated RT protocols, whereas the current study evaluated the outcome with a single protocol (8). The previous study also included a greater number of dogs that received chemotherapy (48%) than the current study (22%) (8). While the current study did not identify an improved outcome following the concurrent administration of chemotherapy, the previous study suggested that the inclusion of chemotherapy led to a statistically significant improvement in MST from 110 to 232 days (8). Given the low numbers of patients that received chemotherapy and the large number of protocols used in the current study, it is difficult to determine if this difference could lead to the observed difference in outcome, but it is considered a reasonable possibility. Additional reasons that may account for the reduced MST in the current study include geographic differences in referral patterns and institutional preferences as to when hypofractionated RT should be administered. As the survival time was measured from the initiation of hypofractionated RT, late referral or delayed initiation of RT could artificially reduce the observed outcome. Overall, the observed MST in this contemporary cohort is considered commensurate with the available literature for palliative-intent treatment of OSA, which is generally regarded to be 1–6 months, depending on the interventions pursued (2).

The current study is also the first to report the outcome of dogs receiving ZOL in addition to hypofractionated RT for the treatment of OSA. The utilization of ZOL in veterinary medicine is relatively recent, with the first use in this cohort not occurring until 2016. Given the improved ease of administration compared to PAM, ZOL has since quickly become the BP of choice at this institution, with no dogs in this cohort receiving PAM after 2016. In the current study, the BP that was administered did not have a significant influence on outcome. Prior studies have suggested a possible anti-tumor or radiosensitizing effect of ZOL, which may be expected to result in improved survival times, but this was not identified in the current study (17, 21, 22). While the current study did not identify an improved outcome with administration of ZOL, the lack of evidence of a worse outcome is considered support for the current trend of replacing PAM with ZOL as the BP of choice in veterinary medicine.

In this population of dogs, only age was identified as a prognostic factor. Dogs that were >9 years of age had a significantly worse prognosis than dogs who were 9 years or younger. This likely represents a combination of several variables, including a greater number of comorbidities in older patients, owner perception of quality of life, and differing preferences for treatment of older dogs. More aggressive tumor behavior being associated with an increase in age is considered less likely but cannot be entirely ruled out. Prior studies have not revealed a consensus as to whether age is a poor prognostic factor for dogs diagnosed with OSA, and previous studies of dogs with OSA treated with palliative RT have not identified age as a significant prognostic factor (5–7, 9, 10, 21–24).

The limitations for this study include possible errors in medical record reporting, lack of histologic or cytologic confirmation in most cases, and the retrospective nature of the study. As this population of dogs underwent palliative-intent treatment, this presented a unique set of challenges due to the lack of a standardized protocol. This resulted in a cohort that did not have routine follow up, received a varying combination of oral analgesics, and received various chemotherapy agents. Additionally, many dogs presented to the institution for hypofractionated RT only, which necessitated a heavy reliance on medical records from referring veterinarians. Due to these limitations, it was not possible to evaluate other indicators of outcome other than survival. Lameness and pain scores were variably reported, and infrequently commented on following RT in this population. Thus, in this population it was not possible to determine if dogs were administered BPs because they were more lame or painful, nor could it be determined if the administration of BPs resulted in a greater magnitude or duration of pain control. Given these limitations, survival time was considered the most objective measure of outcome available. As this is the largest retrospective case series examining palliative-intent hypofractionated RT for canine OSA reported to date, the intention was to keep inclusion criteria as broad as possible in an attempt to provide the most accurate information about outcome for the overall population of dogs that may be candidates for this treatment. While it was possible to identify some patients who had evidence of metastatic disease or pathologic fracture at the time of RT, given the lack of standardized staging these patients represent the minimum number within this population. It is likely that specific subsets of patients, such as those having metastatic disease or pathologic fracture, may have significantly different outcomes compared to the overall population of dogs undergoing these treatments. However, due to the limitations of this study it was not possible to evaluate these subsets.

Given the scant literature available and limitations of the current study, there remains the need for continued evaluation of palliative-intent treatment for canine OSA. Multi-center prospective case-controlled studies should be performed to better define and understand the effect of palliative-intent treatments on outcomes for dogs with appendicular OSA. A prospective clinical trial evaluating survival, pain scores, gait analysis, and owner-perceived quality of life scores with standardized follow-up after receiving hypofractionated RT with or without the addition of BPs would prove invaluable in addressing the remaining questions posed by the current study. Additionally, such a design could further examine recent findings that suggest timing of BP administration may influence survival following irradiation of canine OSA cells (18, 19). In the current study, dogs that received BPs prior to or after RT had a MST of 147 days compared to 114 days for dogs that received BPs concurrent with RT. While not statistically significant, in light of the recent *in vitro* work this suggests an intriguing opportunity to improve outcomes by optimizing timing of palliative-intent treatments.

In conclusion, we have identified no significant impact on survival following a palliative-intent hypofractionated RT protocol with or without the inclusion of BPs. This study suggests no contraindication to the inclusion of BPs in palliative-intent treatment of OSA with hypofractionated RT protocols. This study also suggests there is no difference in outcome between dogs that received ZOL or PAM, supporting the current trend of replacing PAM with ZOL as the BP of choice in veterinary medicine.

## Data Availability Statement

The raw data supporting the conclusions of this article will be made available by the authors, without undue reservation.

## Ethics Statement

Ethical review and approval was not required for the animal study because it was a retrospective study. Written informed consent for participation was not obtained from the owners because this was a retrospective medical record review only. Prior to treatment, informed owner consent was obtained for each patient per the requirements of the veterinary teaching hospital.

## Author Contributions

Data collection performed by BR-M. Data was reviewed and analysis performed by BR-M, DT, and TM. Manuscript preparation was completed by BR-M and was reviewed by all authors. All authors contributed to the article and approved the submitted version.

## Conflict of Interest

The authors declare that the research was conducted in the absence of any commercial or financial relationships that could be construed as a potential conflict of interest.

## Publisher's Note

All claims expressed in this article are solely those of the authors and do not necessarily represent those of their affiliated organizations, or those of the publisher, the editors and the reviewers. Any product that may be evaluated in this article, or claim that may be made by its manufacturer, is not guaranteed or endorsed by the publisher.
